# Veterinary Science1Read before the Missouri State Veterinary Medical Association at St. Louis, October 12, 1894.

**Published:** 1895-01

**Authors:** L. M. Klutts


### VETERINARY SCIENCE.[1]

BY L. M. KLUTTS, V.S.

AFTER a long and somewhat serious consideration I have come to the conclusion to fill my assigned task by describing the agreeable horrors of veterinary science. Unqualified as I am, I approach this wide subject with fear and trembling. I am doubly convinced of one thing: I could not have selected a broader, better, or more fit subject for penning my ungrammatical thoughts for this occasion.

As this honorable body of true and loyal men gaze into my face, you demand of me something of the early struggles of the good and faithful pioneers, who lived a life of success and finally died in the noble cause.

You request me to make honorable mention of the schools and authors of your fancy, knowing, as I do, that we have representation from almost, if not all, the recognized schools in America, and perhaps a few from Europe, France, and Scotland. And allow me to mention right here, that should any of you not be inclined to like the contents of this paper, do not request me to make an apology. There are a great many important things pertaining to veterinary science that I know nothing about, of which I am not liable to write very much.

The science comprises a knowledge of the conformation and structures of all the domesticated animals, including their physiology and individual characteristics, their humane man-

[1] Read before the Missouri State Veterinary Medical Association at St. Louis, October 12, 1894.

agement and utilization, their protection from, and medical and surgical treatment in the diseases and injuries to which they are exposed; their relation to the human family with regard to communicable disorders. In looking over a few statistics, we Americans have to take a back seat. Perhaps, by hard work and perseverance, we will be able to bring up the rear of this progressive science which we have chosen as a profession among many, by which we might be fortunate and successful enough to earn our daily bread.

There is evidence that the Egyptians practised veterinary medicine and surgery in very remote times, but it is not until we turn to the Greeks that we obtain any very definite information with regard to the state of veterinary medicine in antiquity. Aristotle wrote on physiology and comparative anatomy and on the maladies of animals, while many other Greek writers on veterinary medicine are copied by later investigators. Varo (128 B. C.) may be considered the first Roman writer who deals with animal medicine in a scientific spirit. Celsus is also supposed to have written on animal medicine.

The works written by Colemelia on veterinary medicine and surgery also deserve mention. Other editions of the same author, containing sanitary measures for the suppression of contagious diseases, were considered quite good. From the third century onward, veterinary science had a literature of its own and regular practitioners.

Apsyrtus, of Bithinia, perhaps, was the renowned veterinarian of the Roman empire. His professional life seems to have enjoyed a high and well-deserved reputation in his time. Being a keen observer, he distinguished and described a number of diseases which were badly defined by his predecessors. He recognized the contagious nature of several maladies, and prescribed isolation for their suppression.

Not less eminent was Hereocles, the successor of Apsyrtus, whose writings he largely copied, but with improvements and valuable additions, especially in the hygiene and general care of stock.

Veterinary science was undoubtedly nurtured and cradled back among the shepherds. Abraham made himself famous by his examples of breeding for color of different stripes by casting different shadows in drinking pools and placing dazzling objects of different colors in front of his animals at the period of copulation.

Another strong man's life presents itself, by which we learn an important lesson in regard to the bony structures of the mule. We find Samson bound with cords by the strong authority of Israel. Just think of him lying down at the camp of the Philistines. Without a single friend to pity or an atom of law for protection, he bore persecution in silence until he received help from a higher power, when he was enabled to break the cords of bondage and picked up the jawbone of a mule as a weapon of warfare, and the result was an unexpected funeral of over a thousand Philistines.

This is an object lesson by which we learn that things are continually changing; for instance, the Philistines ever since have been just a little jubus of a mule's head, while we fellows down here on the border of the nineteenth century have almost the same superstitious idea of his heels.

Gentlemen, I shall just invite your indulgence for a short time on sacred history, knowing that a few doses administered at regular intervals are both pleasant and stimulating.

This subject of veterinary science demands of me to gather information from earth, sea, and sky, and I will promise to be brief, knowing that my paper will be disagreeably lengthy. The earth upon which we tread is filled with living objects. A large proportion of the sand of the Libian Desert consists of microscopic fossil remains, and the marine sands of the Paris basin are in some localities so full of microscopic forms that it is calculated that a cubic inch of the mass contains sixty thousand.

In the water that we drink animalcules are sometimes visible to the naked eye as moving points, and with the aid of a microscope we are enabled to estimate that a single drop of water will contain many thousands of them.

When we gaze into the aerial space we behold thousands of insects dazzling in the air, not including the mosquitoes in New Orleans, flies in Philadelphia, and bedbugs in Chicago. On closer examination we find that living germs that cause disease are undoubtedly carried in the air from neighborhood to neighborhood.

In my ramble for information to fill my paper I find Jonah is worthy of note. Did any of you fellows ever read the Book of Jonah? I would ask you to hold up your hands if I was not afraid I would skunk you.

I find this historian down along the seashore awaiting the departure of a ship, so he could skip for another country, where he thought he could not hear the commands of his master. We will take him up in our minds for a moment and think of the beautiful picture of imagination he had framed in his mind. What I am going to do when I get over yonder. This ship runs so lovely. Good-by, old country, I am going to leave you. This is just delightful. I am content now; I am going to retire, I can sleep now. I don't know but what he said good-night to the sailors. Another moment, and he is fast asleep. Now think of the terrible storm, and the sailors rushing down there and rousing up this man. Here we will have to cast lots to see who is to be thrown overboard. Next, I see my man at the edge of the ship, in the strong arms of the sailors, and into the water goes our subject, to be swallowed by a large sea monster, or whale. I am undecided what you have learned by this illustration, but I tell you I have learned something of the digestive apparatus of this animal, which links information to veterinary science.

Perhaps the most important era in the history of modern veterinary science commenced with the institution of veterinary schools. France was the first to take an important step in this direction. Bourgelat and Bertin influenced the government to establish a veterinary school at Lyons in 1761, and through the influence of this one many more institutions were founded and became self-sustaining.

Soon we found a school in Austria, and in 1790 I find St. Bel laboring in Europe, and finally formed an institution styled the Veterinary College of London, and from time to time additions were built as the pressing demands became urgent. This school made rapid progress in public estimation under the management of St. Bel. Next in rotation we find veterinary missionaries in India, where very good work was done to prevent contagious disorders. In a short time we find Professor Dick in Scotland, and, with his perseverance and ability, stamped a favorable impression on the minds of his hearers, and up go two veterinary schools, one at Edinburgh and the other at Glasgow. Both are reported as doing good work and in a flourishing condition. The Royal College of Veterinary Surgeons has turned out since 1844 over four thousand registered graduates.

In some of the old countries a veterinary surgeon is recognized by a red cross. Not exactly so in this country; the red coloring matter is generally found in the nose.

2

In 1861 I noticed Dr. Andrew Smith in Toronto, Canada, pleading with the Board of Agriculture, and through his influential demands lectures on this progressive science began, and the institution has proved to be one of the leading schools in the world. They have turned out about two thousand graduates.

Soon we find the University of Pennsylvania coupling on an addition for the purpose of teaching veterinary science, which has proved to be a most successful move. We also find New York taking the fever and building two veterinary institutions. One, known as the American Veterinary College, has got about five hundred graduates out in this great field of practice, and their success proves to us that New York is no bad place to go to seek an education in this great science.

Next in line, Dr. Howard. How shall I call these attentive hearers to Chicago? Come along, boys; let us leave silver and Wall street in New York, and go to the Chicago Veterinary College, a college that I am all wrapped up in; a college that stands as a monument of industry and progress; a college that never allowed its professors to demand help financially; a college that its professors will pluck a fellow if he is not up well on final examination; a college that I shall ever keep fresh in my memory for the good the professors have done and the little some of its graduates have done. Dr. Howard, I shall always remember the 21st of March, 1889, that memorable day down there in Kimball Hall among the sweet music and the happy professors and students we one by one reached out our hands and grasped something entitling us to administer medicine to domesticated animals in cases of emergency. I shall never forget how we swelled up and thought now we were out of bondage.

I shall never forget how much I thought I knew, and how I was cut down when a specimen of insanity asked me a question pertaining to veterinary science that I could not answer. That was a memorable day. I was somewhat reconciled when one of the professors told me that it took a big fool to ask hard questions, and since then I have been as jubus of fools as the Philistines are of mules.

Next in rotation we find the veterinary epidemic all over our land—Detroit, Mich.; Cincinnati, Ohio; St. Paul, Minn.; Washington, D. C.; and, Dr. Wattles, what about Kansas City, Missouri? Yes, our grand old Commonwealth has swung into line, and this State has got an institution that we should all be proud of, and we are proud of it, and it isn't done growing yet, either. I was there a few weeks ago at the opening, and it was very encouraging indeed. I noticed temperance badges dangling on some of the students, and I noticed young men there that represented some of the leading families of our land, which indicated to me that this comparatively young college has an everlasting foundation underneath. Very nearly, if not all, the agricultural colleges have a veterinary department attached.

In going back to Chicago I find the McKillip Veterinary College, one of the finest equipped institutions of learning in the world. Its doors are now open, it being a three-years' course school. The outlook for success is encouraging, indeed.

I will venture to say that no profession has made greater advancement in the last quarter of a century than veterinary science. Where ignorance, superstition, and carelessness once dominated, science and culture now hold sway. The horse doctor, who once held a lance in one hand and mercury in the other, was supposed to fill the bill under any and all circumstances. The order has changed to a class of scientific veterinarians in every way worthy of the noble profession which they have undertaken. We have State and government laws that have been enforced, and the beneficial results are well worthy of note. Through this law we have a foreign meat market, and a number of diseases are held in check. President Cleveland, the angel of purity, in his message to the Secretary of Agriculture, indorsed this law, and urged that every applicant for office should be able to show a diploma from some recognized veterinary college.

Now, in conclusion, I appeal to you practitioners, with the limited ambition of my professional reputation, to avoid smooth, polished promises and flashy advertising cards and newspaper puffs; see to it that you do not promise more than you are able to fulfil; also avoid the association of quacks and empirics, who will present themselves to you with a long list of names of bankers, merchants, and stockmen. See to it, gentlemen, that you do not get misled, but keep in mind that any good citizen, under certain circumstances, will be only too glad to sign a paper to get the false pretender out of his neighborhood. Now, see to it, brother practitioners, that you are not persuaded by your own conscience that in order to build a famous reputation you will have to have a number of badges dangling from your coat, vest, and watch chain, emblems of purity, for the different organizations in your section.

I have the fondest regard for these societies, but I am quite sure they have never taught me how to prognose or diagnose any case in veterinary science. Do not try to serve too many masters. If you are a farmer, strive to be the best in your section. If you are a merchant, be the best in your town. If you are a banker, try to be the best, and if you are so unfortunate as to be a doctor, throw your whole weight into that profession and serve it to the best of your knowledge and ability. Always keep in view that a good reputation (not inherited) is the "Alpha" and "Omega" of many principles. Do not use the word "cure" in connection with your name, but give "honor to whom honor is due." So we will be servants of "vis medicatrix naturæ," and should we prove to be obedient and qualified, our success is sure and our reward will be bountiful.

